
JOURNAL INFORMATION
==============================
NLM Title Abbreviation: ScientificWorldJournal
Journal ID: ScientificWorldJournal
NLM Journal ID: 101131163
Journal ID: TSWJ

The Scientific World Journal

ISSN: 2356-6140
EISSN: 1537-744X
Publisher: Wiley

ARTICLE INFORMATION
==============================
PMCID: PMC5956439
PMID: 15349547
DOI: 10.1100/tsw.2004.72
Article ID: 670141
Article version: 1
Subjects: Original Report

Bilateral Staghorn Calculi in an Eighteen-Month-Old Boy

Netto Jose Murillo B.
Perez Luis M.
Batista Leandro Raus
Roya Stuart A.
Burns John R.
Children's Hospital, University of Alabama at Birmingham, Birmingham, AL, USA
Academic Editor: Anthony Atala

Publication date: 2004
Electronic publication date: 2004 Jun 7
Volume: 4
First page: 249
Last page: 252
Copyright: Copyright © 2004 Jose Murillo B. Netto et al.
Copyright year: 2004
License: This is an open access article distributed under the Creative Commons Attribution License, which permits unrestricted use, distribution, and reproduction in any medium, provided the original work is properly cited.
License URL: https://creativecommons.org/licenses/by/3.0/

==============================
Original Report
*TheScientificWorldJOURNAL* (2004) 4 (S1), 249–252
ISSN 1537-744X; DOI 10.1100/tsw.2004.72

### Bilateral Staghorn Calculi in an Eighteen-Month-Old Boy

Jose Murillo B. Netto, MD, Luis M. Perez, MD, FAAP, Leandro Ruas Batista, MD, Stuart A. Royal, MD, and John R. Burns, MD
*Children's Hospital, University of Alabama at Birmingham, Birmingham, Alabama*

**E-mails**: jmbnetto@uropedjf.com.br

*Previously published in the Digital Urology Journal*

**DOMAIN**: urology

### CASE REPORT

An 18-month-old white boy was referred to our service for bilateral staghorn calculi noted on a routine chest radiograph. He had a history of prematurity resulting in bronchopulmonary dysplasia and hydrocephalus secondary to an intraventricular bleed. These were managed with furosemide administration during the first 3 months of life and a ventriculoperitoneal (VP) shunt. A conventional abdominal radiograph confirmed the presence of bilateral partial staghorn stones (Fig.1a) which on intravenous urography (IVU) completely filled the lower pole moiety of a left duplex system and partially filled the right lower renal pole collecting system (Fig.1b). A screening urine culture was positive for *Proteus mirabilis.* Voiding cystourethrogram was normal. Serum creatinine, calcium, phosphorus, uric acid, and parathyroid hormone levels were normal.

During a single general anesthetic bilateral pyelolithotomies were performed with intraoperative flexible nephroscopy and electrohydrolic lithotripsy of accessible calyces. Bilateral indwelling JJ stents were placed. The follow-up abdominal radiograph revealed bilaterally reduced stone burdens (Fig.2a). Stone analysis revealed 96 % calcium phosphate (90% carbonate form and 6% hydroxyl form) and 4 % protein. Outpatient extracorporeal shockwave lithotripsy (ESWL) was performed during separate sessions on the left and right remnant stone burdens at 1 and 3 months following open surgery, respectively. Each stone burden received 2,000 shocks at 15 kilovolts using the Dornier HM-3 lithotriptor. Following his last ESWL treatment, the child is free of nephrolithiasis by IVU, renal ultrasonography, and conventional radiography performed at 6, 14 and 23 months respectively (Fig.2b). He does have small stone fragments in his retroperitoneum from his open surgery.

### COMMENTS

Although nephrocalcinosis has been frequently reported in infants treated with furosemide, staghorn calculi have rarely been reported in this age group.[1-3] The etiology of nephrocalcinosis associated with furosemide therapy in infants is related to a hypercalciuric state which may be managed by observation, hydration, and thiazide therapy.[4] In addition, the spot urinary calcium-creatinine ratio may aid in

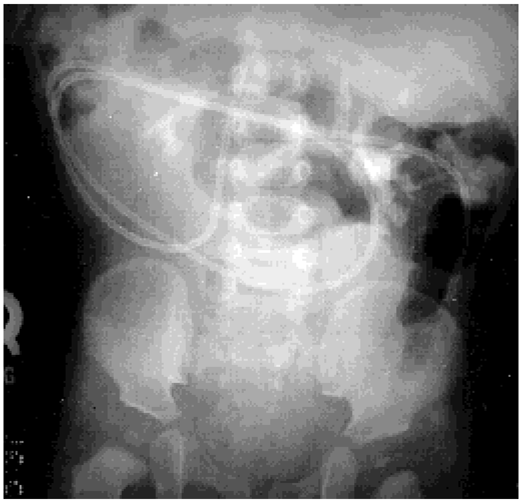

**FIGURE 1a**. Bilateral staghorn calculi. Conventional abdominal radiograph demonstrates a left complete and a right partial staghorn calculi. VP shunt is noted.

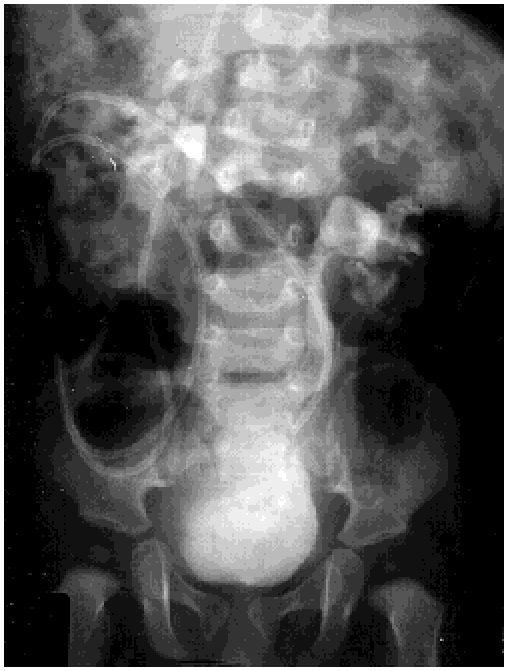

**FIGURE 1b**. IVP showing a left duplex system with the staghorn calculus in the lower renal pole moiety and a single right system with a partial staghorn calculus of the lower renal pole.

predicting which cases have a greater chance of resolution.[5] As in our case, staghorn calculi are generally associated with urinary tract infection with urease-splitting bacteria such as *Proteus* and *Klebsiella.*[1] However, although our child had a complete and partial staghorn calculi as well as *Proteus* bacteriuria, his stone analysis revealed mainly a stone composed of calcium phosphate (apatite) rather than magnesium ammonium phosphate (struvite).[1]

The treatment of staghorn calculi is more technically demanding in infants than older children or adults due to size of the kidneys, an increased chance of renal artery thrombosis secondary to aggressive renal mobilization, and more difficult percutaneous access with standard equipment. Therefore, more than one surgical intervention may be required to eradicate a large renal stone burden in an infant. Similar to the adult population, the treatment options include anatrophic nephrolithotomy, pyelolithotomy, percutaneous nephrolithotomy, and ESWL.[1, 3] We believe that it is critical to completely eradicate significant stone burdens in any child in the hope to preserve long-term renal function. Recently, the use of an 11 French (F) peel-away access sheath in combination with electrohydraulic lithotripsy or

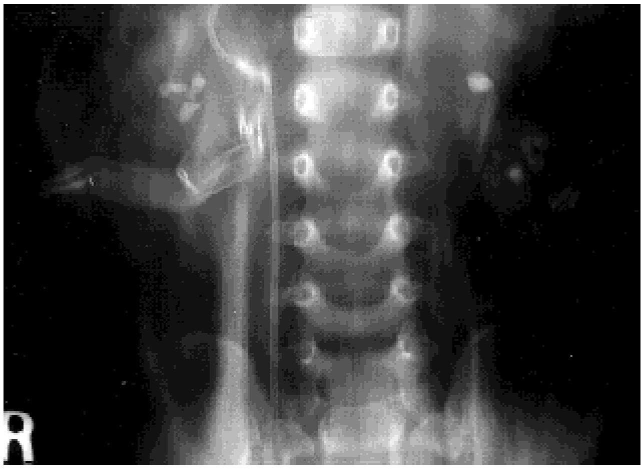

**FIGURE 2a**. Conventional radiograph demonstrates reduced stone burden following bilateral open surgery. Bilateral penrose drains and JJ ureteral stents in place.

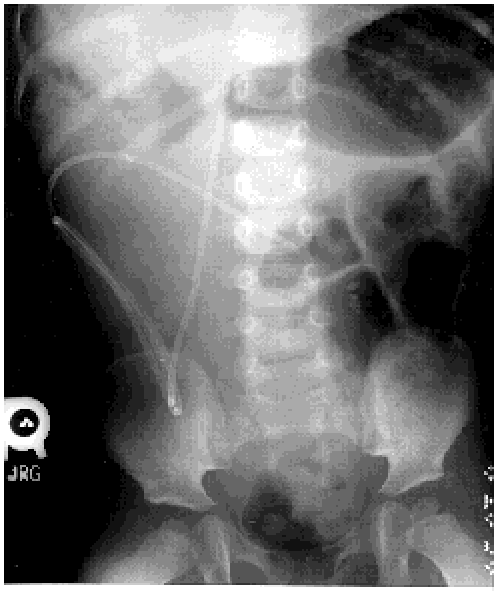

**FIGURE 2b**. Conventional radiograph after bilateral ESWL showing resolution of calculi. The calcifications seen are located in the retroperitoneum outside the collecting system, as a result stone fragment spillage during the open surgery.

Holmium-YAG laser has made the percutaneous nephrolithotomy more attractive in infants and small children than open surgery combined with ESWL.[6] Previously, 24 to 30 F working percutaneous nephrostomy sheaths had been used in children which in theory would result in significant trauma to the infant's kidney.[6]

### REFERENCES

1. Segura JW. Staghorn calculi. Urol Clin North Am 1997; 24 (1): 71-80.
2. Ezzedeen F, Adelman RD, Ahlfors CE. Renal calcification in preterm infants: pathophysiology and long-term sequelae. J Ped 1988; 113 (3): 532-539.
3. Burns JR, Joseph DB. Combination therapy for a partial staghorn calculus in an infant. J Endourol 1993; 7 (6): 469-471.
4. Hufnagle KG, Khan SN, Penn D, Cacciarelli A, Williams P. Renal calcifications: a complication of long-term furosemide therapy in preterm infants. Pediatrics 70: 360-363, 1988.
5. Pope JC, Trusler LA, Klein AM, Walsh WF, Yared A, Brock JW. The natural history of nephrocalcinosis in premature infants treated with loop diuretics. J Urol 156: 709-712, 1996.
6. Jackman SV, Hedican SP, Peters CA, Docimo SG. Percutaneous nephrolithotomy in infants and preschool age children: experience with a new technique. Urology 52: 697-701, 1998.

**This article should be referenced as follows:**

Netto, J.M.B., Perez, L.M., Batista, L.R., Royal, S.A., and Burns, J.R. (2004) Bilateral staghorn calculi in an eighteen-month-old boy. *TheScientificWorldJOURNAL* **4 (S1)**, 249–252.

**Handling Editor:**

Anthony Atala, Principle Editor for *Urology* — a domain of *TheScientificWorldJOURNAL*.
